# Magnetic phase separation in double layer ruthenates Ca_3_(Ru_1−*x*_Ti_*x*_)_2_O_7_

**DOI:** 10.1038/srep19462

**Published:** 2016-01-14

**Authors:** Jin Peng, J. Y. Liu, J. Hu, Z. Q. Mao, F. M. Zhang, X. S. Wu

**Affiliations:** 1 Collaborative Innovation Center of Advanced Microstructures, Lab of Solid State Microstructures, School of Physics, Nanjing University, Nanjing 210093, P. R. China; 2 Department of Physics and Engineering Physics, Tulane University, New Orleans, Louisiana 70118, USA

## Abstract

A phase transition from metallic AFM-b antiferromagnetic state to Mott insulating G-type antiferromagnetic (G-AFM) state was found in Ca_3_(Ru_1−*x*_Ti_*x*_)_2_O_7_ at about *x* = 0.03 in our previous work. In the present, we focused on the study of the magnetic transition near the critical composition through detailed magnetization measurements. There is no intermediate magnetic phases between the AFM-b and G-AFM states, which is in contrasted to manganites where a similar magnetic phase transition takes place through the presence of several intermediate magnetic phases. The AFM-b-to-G-AFM transition in Ca_3_(Ru_1−*x*_Ti_*x*_)_2_O_7_ happens through a phase separation process in the 2–5% Ti range, whereas similar magnetic transitions in manganites are tuned by 50–70% chemical substitutions. We discussed the possible origin of such an unusual magnetic transition and compared with that in manganites.

Transition metal oxides (TMOs), especially those possessing perovskite structures, have been attracting enormous attention since the discovery of high-temperature superconductivity in cuprates^1^,^2^ and colossal magnetoresistivity (CMR)in manganites^3^,^4^,^5^,^6^,^7^. These systems provide a fertile ground for the study of some fundamental issues in condensed matter physics, *e.g*. electron-electron interactions. Moreover, TMOs have substantial promises for advanced technological applications, such as superconducting devices^8^,^9^, spintronics^10^, ferroelectric memories^11^,^12^ and so on. The most significant characteristic of TMOs is that they exhibit a broad spectrum of electronic and magnetic properties. The rich exotic phenomena in TMOs can be attributed to the complex interplay among charge, spin, lattice and orbital degrees of freedom. These interactions result in a soft electromagnetic state which can be easily modified by external perturbations (*e.g*. electric/magnetic field, pressure and temperature)^13^. Ruddlesden-Popper (RP) series ruthenates are typical TMOs with perovskite structures. The 4*d* electron orbitals of Ru are more extended than 3*d* orbitals, the interplay of charge, spin, lattice and orbital degrees of freedom in ruthenates is thus stronger compared to 3*d* TMOs, which results in a rich variety of exotic properties in ruthenates. The exotic phenomena observed in ruthenates thus far include spin-triplet superconductivity in Sr_2_RuO_4_ 14,15,16, field-tuned electronic nematicity in Sr_3_Ru_2_O_7_ 17,18,19,20, itinerant ferromagnetism in SrRuO_3_ 21, antiferromagnetic (AFM) Mott insulating state in Ca_2_RuO_4_ 22,23, quasi-two-dimensional metallic state with an AFM order in Ca_3_Ru_2_O_7_ 24,25, and paramagnetic (PM) ‘bad’ metallic state in CaRuO_3_ 26. Furthermore, since ruthenates have more extended *d* orbitals, weaker on-site Coulomb repulsion energy and stronger *p-d* orbital hybridization than 3*d* TMOs, their physical properties are more sensitive to the perturbations such as magnetic field and chemical doping.

In the present, we explore the underlying physics of the magnetic transition discovered in Ti doped Ca_3_Ru_2_O_7_ 27,28. Undoped Ca_3_Ru_2_O_7_ shows an antiferromagnetic (AFM) transition at 56 K, which is then followed by a metal-insulator transition (MIT) at 48 K^24^,^25^. While Photoconductivity and Raman spectroscopy measurements reveal a charge gap opening associated with the MIT^29^,^30^, angle-resolved photoemission spectroscopy measurements (ARPES) prove small metallic Fermi pockets survive from the MIT^31^. This explains the reentrance of quasi-2D metallic state below 30 K^25^. The AFM state below 56 K is characterized by ferromagnetic (FM) bilayers coupled antiferromagnetically along the *c* axis. The spin direction switches from the *a*-axis for *T*_MIT_ < *T* < *T*_N_ to the *b*-axis for *T* < *T*_MIT_^24^,^25^,^32^,^33^. Here we use AFM-a and AFM-b to denote these two magnetic states respectively, following the notations used in the previous reports^32^,^33^. The schematic diagram of AFM-b magnetic structure is shown in Fig. 1b. With about 3% Ti doping, the magnetic ground state switches to a G-type AFM state which is characterized by the nearest-neighbor AFM coupling for both the in-plane and *c*-axis directions as shown in Fig. 1c, in sharp contrast to the intra-bilayer FM coupling in the AFM-a or AFM-b state. The spin of the G-AFM state points to the direction that is ~30° to *b* axis, ~60° to *a* and *c* axis, *i.e* around the body diagonal direction^27^. The G-AFM state is accompanied by Mott insulating properties, which is distinct to metallic transport properties in the AFM-a or AFM-b state.

When the Ti content is in the range of 0% and 3%, the system may show complex magnetic transitions with temperature decreasing. An intermediate magnetic (IM) phase in a narrow temperature range between AFM-a and AFM-b is found, which exhibits an incommensurate component^27^,^28^. Moreover, no change in space group symmetry was detected across the MIT in the limitation of XRD and neutron measurements despite the changes in lattice parameters^27^. We also focus on the issue: how does a metallic AFM-b state evolve to an insulating G-AFM state? In manganites, a similar magnetic transition takes place through several intermediate distinct magnetic phase, such as A-AFM (*i.e*. FM layers coupled antiferromagnetically), C-AFM (*i.e*. FM rods coupled antiferromagnetically), CE-AFM (*i.e*. Zigzag FM chains coupled antiferromagnetically)^34^,^35^,^36^,^37^. There is no intermediate magnetic phases is found in Ca_3_(Ru_1−*x*_Ti_*x*_)_2_O_7_ between the AFM-b and G-AFM phases. The new phase diagram shows the AFM-b-to-G-AFM transition occurs through a phase separation process within a narrow composition range (i.e. 2–5% Ti). This finding highlights the comparable energy scale between the intra-bilayer FM and nearest-neighbor AFM coupling in Ca_3_Ru_2_O_7_.

## Results

**Figure 1:**
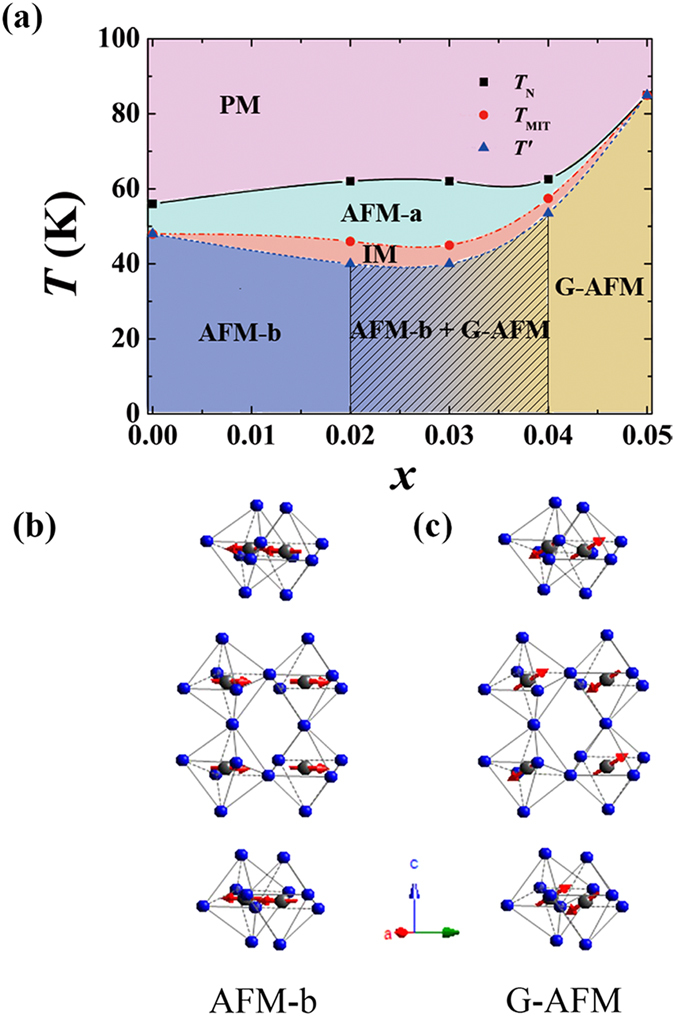
(**a**) Magnetic phase diagram of Ca_3_(Ru_1−*x*_Ti_*x*_)_2_O_7_ 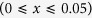. Magnetic phase PM, AFM-a, IM, AFM-b and G-AFM are represented by different color and labels. Temperature driven magnetic phase transitions are marked by solid or dash lines. Doping induced magnetic phase transition from AFM-b to G-AFM through the phase separation are illustrated by a shadow with gradient color background; (**b**) AFM-b magnetic structure; (**c**) G-AFM magnetic structure.

We present the magnetic phase diagram constructed through magnetization measurements on Ca_3_(Ru_1−*x*_Ti_*x*_)_2_O_7_ single crystals in Fig. 1a. Unlike our previously-reported phase diagram^28^ which shows the evolution of magnetic structure in a wide composition range, the current phase diagram is focused on the magnetic phase separation region near the critical Ti concentration. Our goal of establishing such a detailed phase diagram is to examine if there exists any other intermediate magnetic phases between the AFM-b and G-AFM phase. This phase diagram shows that the Ti-doping induced magnetic ground state transition from the AFM-b to G-AFM phase takes place through a phase separation process within a narrow composition region, as illustrated by the shadow with gradient color background in Fig. 1a. The G-AFM phase (yellow color) begins to appear in the *x* = 0.02 sample as a minor phase. Its volume fraction gradually increase from *x* = 0.02 to 0.04. No intermediate phases such as A-AFM and C-AFM were found. Moreover, we also found that the temperature range of AFM-a and IM phases, which occur prior to the presence of AFM-b phase, shrinks as the ground state changes from AFM-b to G-AFM. Experiments for establishing this magnetic phase diagram are described in details below.

**Figure 2:**
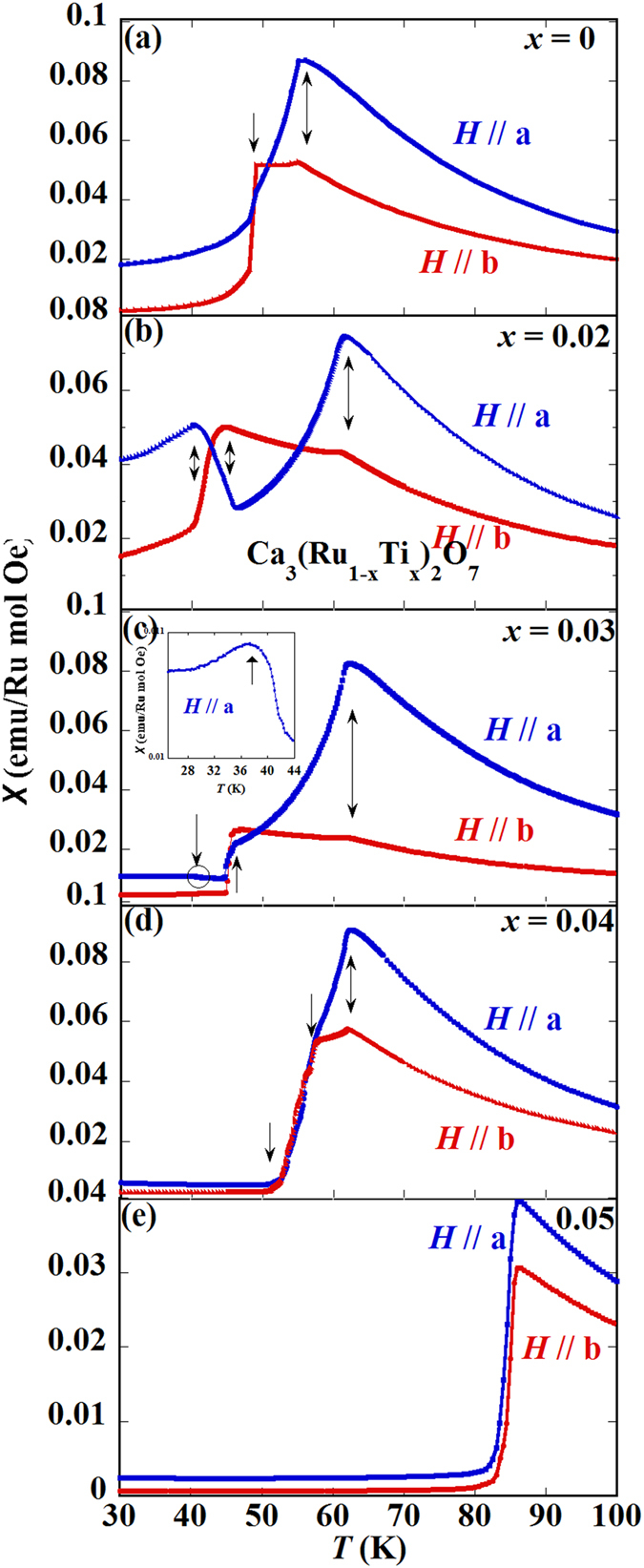
Magnetic susceptibility vs. temperature measured with zero field cooling (ZFC) histories under an external field of 5000 Oe applied along *a* and *b* axes for (**a**) Ca_3_Ru_2_O_7_; (**b**) *x* = 0.02; (**c**) *x* = 0.03; (**d**) *x* = 0.04 and (**e**) *x* = 0.05.

Magnetic susceptibilities versus temperature for Ca_3_(Ru_1−*x*_Ti_*x*_)_2_O_7_ (*x* = 0, 0.02, 0.03, 0.04 and 0.05) are presented in Fig. 2. The data of the pristine compound Ca_3_Ru_2_O_7_ are consistent with previous reports^24^,^25^,^38^. Specially, the data collected with the external field (5000 Oe) applied along the *a*-axis (*H*//a) peaks at *T*_N_ ~ 56 K, while the data measured with the field applied along the *b*- axis (*H*//b) exhibits two anomalies at *T*_N_ and *T*_MIT_ (Fig. 2a). For the *x* = 0.02 sample (Fig. 2b), when *H*//a, the susceptibility curve peaks at 62 K which is the Néel temperature, then it experiences a valley at 46 K, and another peak at 40 K. When *H*//b, the susceptibility data shows a kink at 62 K and a peak at 46 K, followed by a knee point at 40 K. For *x* = 0.03 (Fig. 2c), while the *H*//*b* curve is similar to that of the *x* = 0.02 sample, the lower temperature peak in the *H*//a curve, which is seen at 40 K for *x* = 0.02, becomes much smaller, as indicated by a circle in Fig. 2c. The enlargement of this feature is shown in the inset of Fig. 2c. The magnetic susceptibility data of the *x* = 0.03 sample for the external field along both in-plane *a*/b and out-of-plane *c* direction were previously reported^27^. Those reported data are consistent with the data presented here in general, except for a stronger low temperature peak in the previous data. This may be caused by slight fluctuations of Ti content. In contrast, for the *x* = 0.04 (Fig. 2d), the *H*//*a* and *H*//*b* curves have similar shape. As compared to the *x* = 0.03 sample, three anomalous temperatures move close to each other, as indicated by arrows, with the anomalous feature at the lower temperature side barely observable. For *x* = 0.05 (Fig. 2e), the system exhibits only a single magnetic transition, with the magnetization following similar temperature dependences between the *a* and *b* axes. Overall, with the increase of Ti content, the three magnetic anomalies move closer to each other and finally merge into a single magnetic transition for *x* = 0.05.

**Figure 3:**
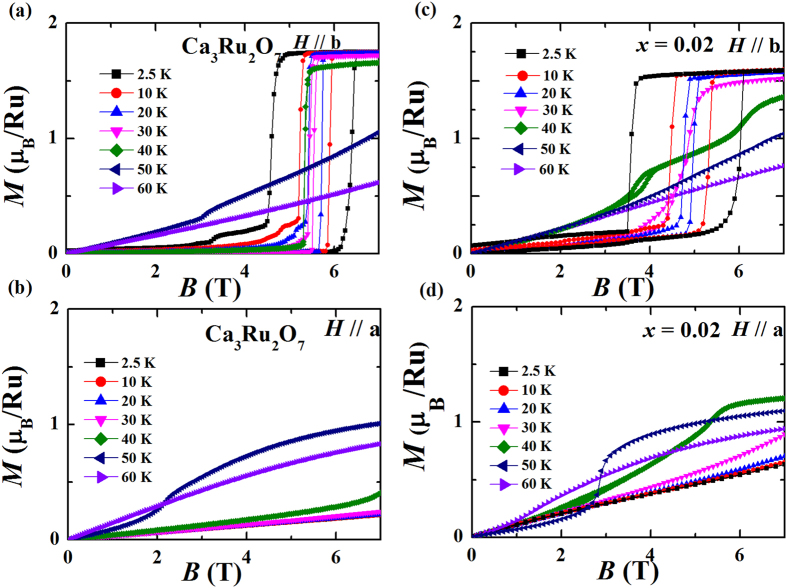
Isothermal magnetization data for (**a**) Ca_3_Ru_2_O_7_, *H*//b; (**b**) Ca_3_Ru_2_O_7_, *H*//a; (**c**) Ca_3_(Ru_1−*x*_Ti_*x*_)_2_O_7_ (*x* = 0.02), *H*//b; and (**d**) Ca_3_(Ru_1−*x*_Ti_*x*_)_2_O_7_ (*x* = 0.02), *H*//a at typical temperatures from 2.5 K to 60 K.

The isothermal magnetization curves at different temperatures for parent compound Ca_3_Ru_2_O_7_ and the *x* = 0.02 samples are shown in Fig. 3. Ca_3_Ru_2_O_7_ undergoes a metamagnetic transition at about 6 T with a wide hysteresis in the sweep-up and –down processes at 2.5 K for *H*//b (Fig. 3a). This metamagnetic transition was found to originate from a transition from an AFM state with anti-parallel spin alignment between bilayers (*i.e*. AFM-b) to a canted AFM state (CAFM) with the spin orientation between adjacent layers differing by ~20°^ ^33. As the temperature increases, both transition fields and hysteresis loops become smaller due to the increased thermal fluctuations. Dramatic changes of *MH* curves for *H*//*b* happen between 40 K and 50 K, from a metamagnetic transition shape to an almost linear field dependence. For *H*//*a* (Fig. 3b), *M*(*H*) curves show linear dependence on field below 40 K. The magnetization value at 7 T for *H*//*b* is much larger than that for *H*//*a* until the temperature reaches 50 K, which is consistent with previous finding that there is an easy axis switching at *T*_MIT_ = 48 K^32^,^33^. Our *M*(*H*) data for Ca_3_Ru_2_O_7_ are consistent with the previous reports^39^.

The *M*(*H*) curves for sample with *x* = 0.02 are presented in Fig. 3c,d, which show similar feature as the pristine compound. However, we find that the difference between *H*//*b* and *H*//*a* is smaller than that for Ca_3_Ru_2_O_7_ based on the following observations: 1) the saturated moment value for *H*//*b* at 2.5 K (~1.6 *μ*_B_/Ru) is smaller than that for Ca_3_Ru_2_O_7_ (~1.8 *μ*_B_/Ru), whereas the magnetic moment for *H*//*a* (~0.65 *μ*_B_/Ru) at 2.5 K and 7 T is obviously larger than that for Ca_3_Ru_2_O_7_ (~0.25 *μ*_B_/Ru). 2) The metamagnetic transition for *H*//*b* begins to broaden above 30 K for *x* = 0.02, while in Ca_3_Ru_2_O_7_ the broadening of the transition occurs above 40 K. More specifically, at 40 K, Ca_3_Ru_2_O_7_ still shows a first order metamagnetic transition at about 5 T; in contrast, for the *x* = 0.02 sample, two step transitions were clearly observed at 6 T and 3.5 T, respectively. 3) For Ca_3_Ru_2_O_7_, the magnetization value at 7 T for *H*//*b* is larger than that for *H*//*a* below 50 K. For the *x* = 0.02 sample, the magnetization value at 7 T for *H*//*a* surpasses that for *H*//*b* at around 40 K. The softening of the AFM-b ground state might be associated with the appearance of minor G-AFM phase in the *x* = 0.02 sample which will be discussed in details later.

**Figure 4:**
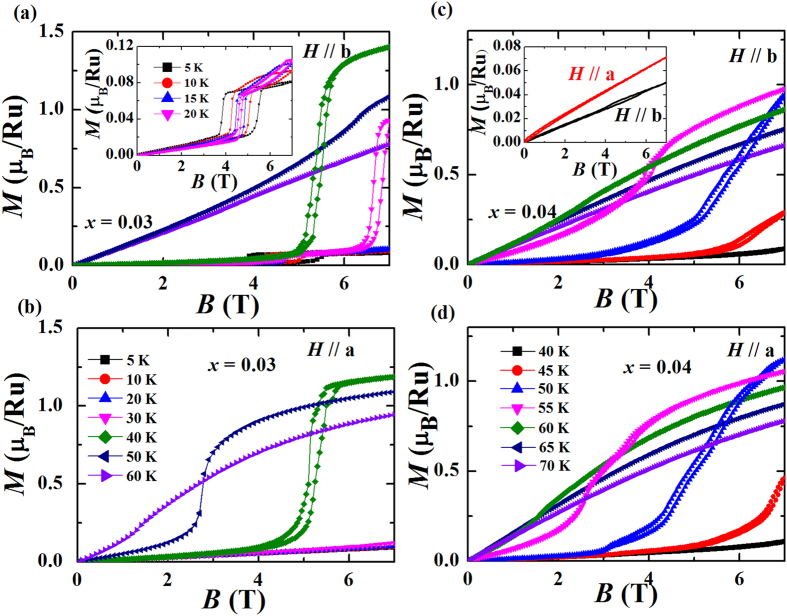
Isothermal magnetization data for Ca_3_(Ru_1−*x*_Ti_*x*_)_2_O_7_ (*x* = 0.03) with external field (**a**) *H*//*b* and (**b**) *H*//*a* at typical temperatures from 5 K to 60 K. Inset in (**a**) Isothermal magnetization data for Ca_3_(Ru_1−*x*_Ti_*x*_)_2_O_7_ (*x* = 0.03) with field applied along *b*-axis at 5 K, 10 K, 15 K and 20 K. Isothermal magnetization data for Ca_3_(Ru_1−*x*_Ti_*x*_)_2_O_7_ (*x* = 0.04) with external field (**c**) *H*//*b* and (**d**) *H*//*a* at typical temperatures from 40 K to 70 K. Inset in (**c**) Isothermal magnetization data taken at 2 K for Ca_3_(Ru_1−*x*_Ti_*x*_)_2_O_7_ (*x* = 0.04).

For *x* = 0.03, the system enters the G-AFM region. Our previous elastic neutron measurements established the ground state of this composition to be a major G-AFM phase and a minor AFM-b phase^27^. As shown in Fig. 4a,b, a first-order metamagnetic transition at ~6 T, corresponding to the AFM-b-to-CAFM transition, can be observed in isothermal magnetization measurements with *H*//b (Fig. 4a inset). This feature is similar to what happened in Ca_3_Ru_2_O_7_. However, the saturated magnetic moment is only ~0.08 *μ*_*B*_/*Ru* above the transition field, which is far away from the expected value of the fully polarized spin moment of Ru^4+^ (S = 1, *M*_s_ = 2 *μ*_*B*_/*Ru*) or the saturated moment measured experimentally for Ca_3_Ru_2_O_7_ (~1.8 *μ*_*B*_/*Ru*). This result indicates that the volume fraction of AFM-b phase is quite small (~4% from estimation). With temperature increasing, the hysteresis of the metamagnetic transition for AFM-b phase is reduced due to enhanced thermal fluctuations. Meanwhile, a second transition at a higher field appears. This transition should be attributed to the polarization of major G-AFM phase. It moves into the equipment capable region 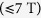 for *T* > 30 K as the transition field decreases with temperature increasing. The saturated moment for *T* = 35 K is ~1.5 *μ*_*B*_/*Ru*, three quarters of the fully polarized spin moment of Ru^4+^. *M* (*H*) for *H*//*a* at low temperatures (<30 K) is almost linear to the field, up to 7 T (see Fig. 4b). The *M*(*H*) curves look similar between *H*//*a* and *H*//b for 30 K < *T* < 50 K, which can be attributed to the canted spin configuration in G-AFM phase: the spin is pointed to the direction which is ~30° to *b* axis, ~60° to *a* and *c* axis^27^. For temperatures above 50 K, the magnetic moments at 7 T for *H*//a are larger than those for *H*//b, indicating the magnetic easy axis switches from the *b*-axis for the major G-AFM phase (*T* < *T*_MIT_) to the *a*-axis for the AFM-a phase (*T*_MIT_ < *T* < *T*_N_).

When Ti doping level is increased to 4%, at 2 K, we still observe a trace of the metamagnetic transition arising from the polarization of AFM-b phase when the field is applied along the *b*-axis (Fig. 4c, inset). This feature indicates a negligible amount of AFM-b phase at the ground state of the 4% Ti doped sample. The metamagnetic transition is quickly submerged by the thermal fluctuation. We note the *M*(*H*) curves for *H*//*b* and *H*//*a* are quite similar to each other (Fig. 4c,d) and the polarization field of the G-AFM phase decreases with temperature increasing.

**Figure 5:**
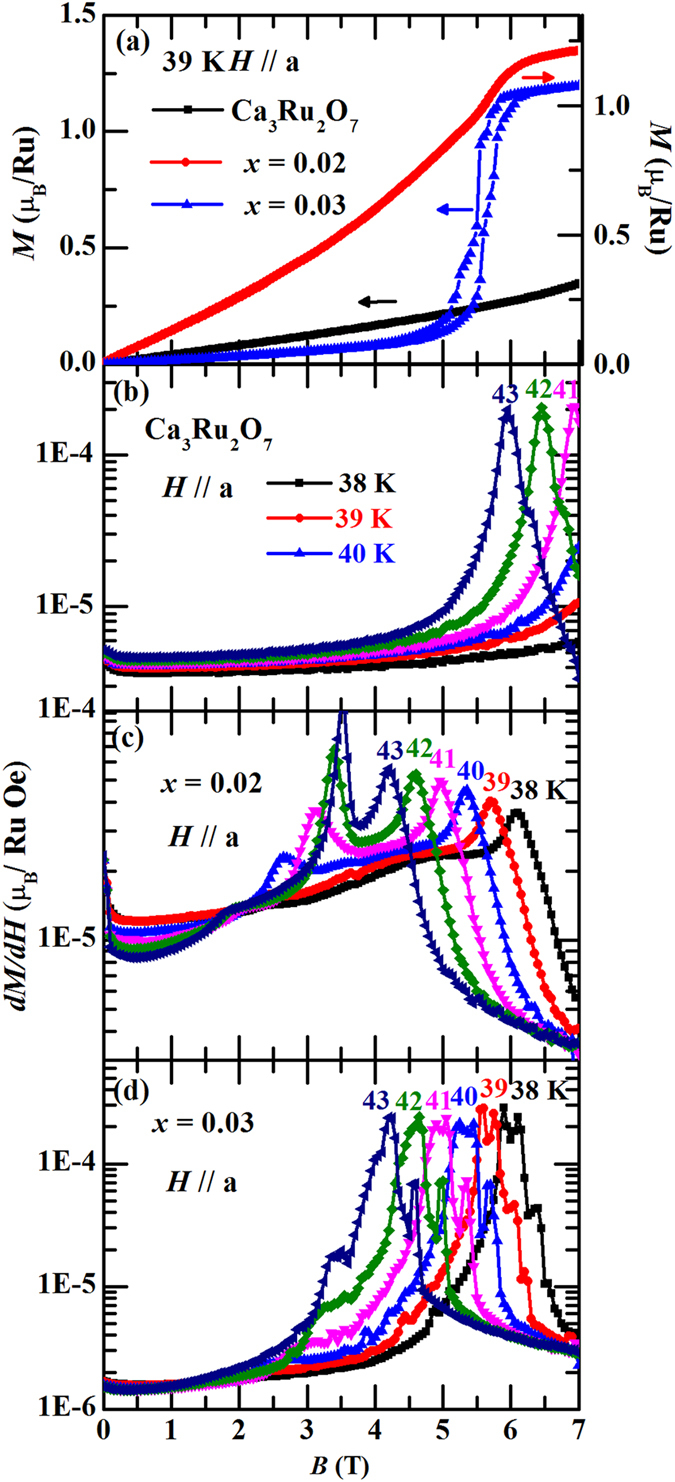
(**a**) Isothermal magnetization for Ca_3_Ru_2_O_7_, Ca_3_(Ru_1−*x*_Ti_*x*_)_2_O_7_ (*x* = 0.02) and Ca_3_(Ru_1−*x*_Ti_*x*_)_2_O_7_ (*x* = 0.03) with *H*//*a* at *T* = 39 K; (**b**) Derivative of isothermal magnetization with respect to magnetic field for Ca_3_Ru_2_O_7_ at 38 K − 43 K; (**c**) Derivative of isothermal magnetization with respect to magnetic field for Ca_3_(Ru_1−*x*_Ti_*x*_)_2_O_7_ (*x* = 0.02) at 38 K − 43 K; (**d**) Derivative of isothermal magnetization with respect to magnetic field for Ca_3_(Ru_1−*x*_Ti_*x*_)_2_O_7_ (*x* = 0.03) at 38 K − 43 K.

The above results of *M*(*H*) measurements for the samples with a major G-AFM phase in the ground state 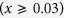 clearly indicate that magnetic phase separation exists not only in the *x* = 0.03 sample but also in the *x* = 0.04 sample. For the *x* = 0.02 sample whose ground state is dominated by the AFM-b phase, we believe the G-AFM phase exists as a minor phase though it is more difficult to be resolved. When the external field is applied along the *b*-axis, the polarization of the minor G-AFM phase in the *x* = 0.02 sample, if it exists, would be easily submerged by the polarization of major AFM-b phase since that the polarization field of the G-AFM phase is much larger than that of the AFM-b phase. When the external field is applied along the *a*-axis, the polarization field for the AFM-b phase is strongly enhanced (~15 T at 0.4 K)^39^. In this case, it is relatively easy to find the imprint of G-AFM phase. By carefully comparing the *M*(*H*) curves of *H*//*a* between Ca_3_Ru_2_O_7_, the *x* = 0.02 and 0.03 samples, we found a trace of metamagnetic transition in the *x* = 0.02 sample at about 5.9 T for *T* = 39 K (Fig. 5a), which can be attributed to the polarization of the G-AFM phase. This argument is based on the fact that due to the polarization of the G-AFM phase, the *x* = 0.03 sample, which involves G-AFM phase as its major phase, also exhibits a magnetic polarization near 5.9 T at 39 K for H//a. Further, we plot the derivative of magnetization vs. external magnetic field for Ca_3_Ru_2_O_7_, the *x* = 0.02, and *x* = 0.03 samples in Fig. 5b–d. The peaks in derivative curves clearly reflect the metamagnetic transition fields. The peaks’ positions for the *x* = 0.02 sample are consistent with those for *x* = 0.03 at corresponding temperatures. These observations suggest the existence of minor G-AFM phase in the *x* = 0.02 sample.

**Figure 6:**
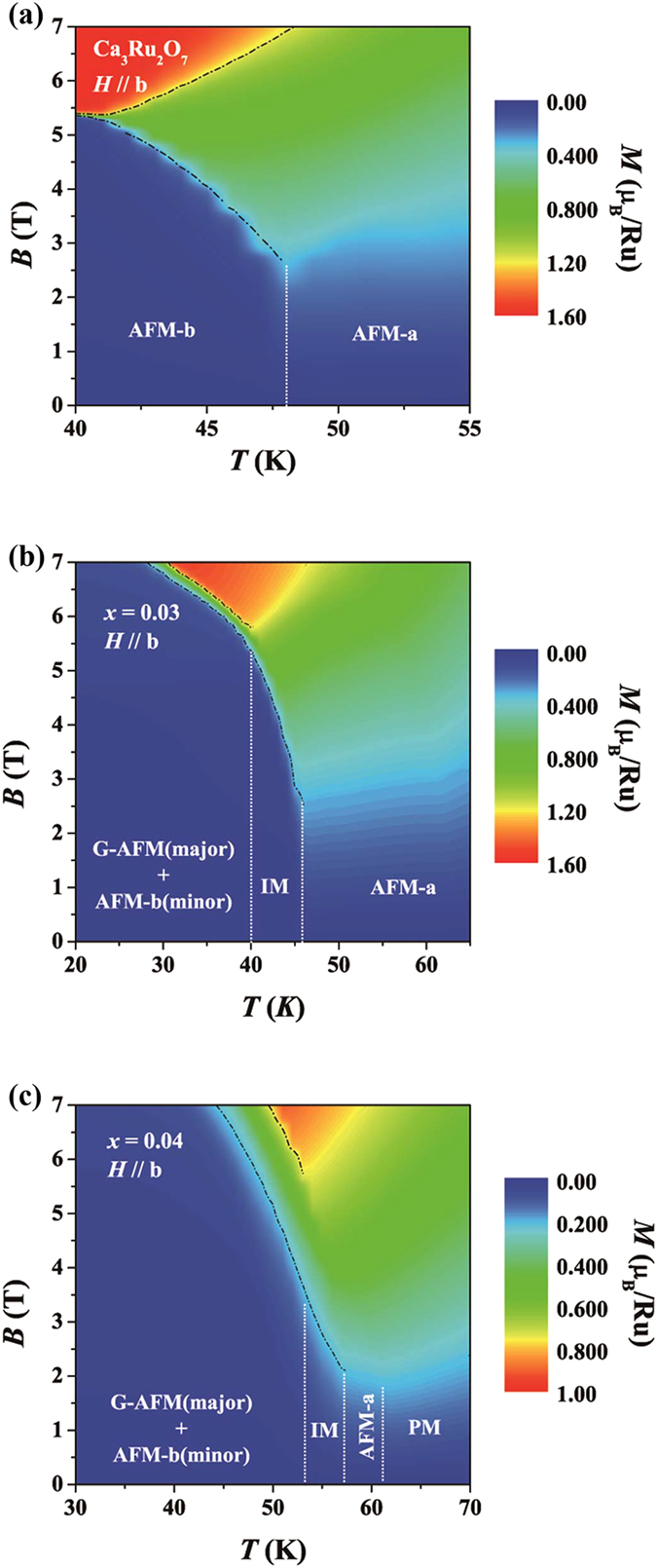
*H-T* phase diagrams for *H*//*b* of (**a**) Ca_3_Ru_2_O_7_, (**b**) Ca_3_(Ru_1−*x*_Ti_*x*_)_2_O_7_ (*x* = 0.03) and (**c**) Ca_3_(Ru_1−*x*_Ti_*x*_)_2_O_7_ (*x* = 0.04). Contour maps are plotted based on magnetization strength (μ_B_/Ru). Major (or pure) and minor magnetic phases are labeled. Temperature driven phase transitions are marked by white dot lines. Fields driven phase transitions are marked by black dash lines.

To gain further insights into the magnetic transitions of Ca_3_(Ru_1−*x*_Ti_*x*_)_2_O_7_, we have also established *H-T* phase diagrams for Ca_3_Ru_2_O_7_, the *x* = 0.03 and 0.04 samples in terms of contour plots of magnetization *M(H)* (*H*//*b*) of these samples (Fig. 6a–c). These phase diagrams allow us to examine the spin flip and flop transitions of the AFM-b and G-AFM states driven by magnetic fields. In Ca_3_Ru_2_O_7_, the field-tuned transition from the AFM-b phase to the CAFM phase is a first-order transition at temperatures below 41 K, but it becomes a second order/crossover transition for *T* > 41 K, as reflected in the bifurcation of the dashed phase boundary line above 41 K (Fig. 6a). When the magnetic phase changes from AFM-b to AFM-a phase at high temperatures (>48 K), the field driven polarization process vanishes since the spin easy axis switches to the *a*-axis. For the *x* = 0.03 and 0.04 samples, we observed a polarization process involving the presence of an intermediate phase. The system transits from a mixed state composed of the major G-AFM phase and the minor AFM-b phase to a partially polarized phase (represented by the green color between two dashed phase boundary lines in Fig. 6b,c) and finally to a polarized state (red color). The field range of the partially polarized phase is ~0.25 T and 1 T for *x* = 0.03 (Fig. 6b) and 0.04 (Fig. 6c) respectively. The polarization field decrease gradually with temperature increasing. The nature of such a partially polarized, intermediate phase is yet to be understood.

## Discussions

Magnetic phase transitions and phase separations are common features in 3*d* transition metal oxides, especially in manganites such as La_1−*x*_Ca_*x*_MnO_3_, Nd_1−*x*_Sr_*x*_MnO_3_ and La_2−2*x*_Sr_1+2*x*_Mn_2_O_7_. If we compare the magnetic phase transition of Ca_3_(Ru_1−*x*_Ti_*x*_)_2_O_7_ as shown in Fig. 1 with those of manganites, we not only note some similarities and but also find significant discrepancies between these two systems. In manganites, magnetic phase transitions from FM to G-AFM are ubiquitous. However, these two magnetic states are normally connected by one or more intermediate magnetic phases and magnetic phase separations are also emerging in some intermediate phases. For example, in La_1−*x*_Ca_*x*_MnO_3_, the FM metallic phase and G-AFM insulating phase are separated by a CE-AFM phase (*i.e*. Zigzag FM chains coupled antiferromagnetically) and a mixed magnetic state composed of FM and CE-AFM phases^36^,^37^. In Nd_1−*x*_Sr_*x*_MnO_3_, however, the intermediate phases include the CE-AFM, A-AFM (*i.e*. FM layers coupled antiferromagnetically) and C-AFM (*i.e*. FM rods coupled antiferromagnetically) phases; phase coexistence of FM, A-AFM and CE-AFM also occurs^35^. In double layer manganites La_2−2*x*_Sr_1+2*x*_Mn_2_O_7_, which is isostructural to Ca_3_(Ru_1−*x*_Ti_*x*_)_2_O_7_, the system evolves from an AFM state consisting of FM bilayers (similar to the AFM-b state shown in our phase diagram in Fig. 1a) to several intermediate phases, including FM, CAFM(canted AFM), A-AFM and C-AFM, before it reaches the G-AFM phase^34^.

One similarity between Ca_3_(Ru_1−*x*_Ti_*x*_)_2_O_7_ and manganites is manifested in the observation of AFM-b and G-AFM states, which are present in both systems. Since the AFM-b state corresponds to a magnetic state with FM bilayers coupled antiferromagnetically along the *c*-axis; the intra-bilayer nearest neighboring Ru-Ru coupling is FM and its coupling strength should be much stronger than the inter-bilayer AFM coupling strength, which is evidenced by the fact that Ca_3_Ru_2_O_7_ has a positive Curie-Weiss temperature (~80 K) despite an inter-bilayer AFM order^25^,^40^. Therefore, the AFM-b state can be approximately viewed as being analogous to the metallic FM state seen in manganite. The other similarity is that we observed magnetic phase separation in the transition from the AFM-b to G-AFM phase. One remarkable discrepancy from manganite is that in Ca_3_(Ru_1−*x*_Ti_*x*_)_2_O_7_ we did not find any intermediate magnetic phases such as C-AFM and CE-AFM between the AFM-b and G-AFM phases. Another striking difference is that the Ca_3_(Ru_1−*x*_Ti_*x*_)_2_O_7_ system requires only a few percent Ti doping to realize the AFM-b-to-G-AFM transition (Fig. 1a), while manganites require a few ten percent chemical substitutions to drive the FM-to-G-AFM transition, *e.g*. >50% Ca/Sr substitution for La/Nd for La_1−*x*_Ca_*x*_MnO_3_/Nd_1−*x*_Sr_*x*_MnO_3_ 35,36,37, ~70% Sr substitution for La for La_2−2*x*_Sr_1+2*x*_Mn_2_O_7_ 34.

The mechanism of the magnetic transition tuned by Ti doping in Ca_3_(Ru_1−*x*_Ti_*x*_)_2_O_7_ is distinct from those of the chemical-substitution induced magnetic transitions in manganite in several aspects. The magnetic transitions in manganites are generally attributed to the competition between the double-exchange FM interaction and superexchange interactions^4^,^7^,^13^. The double-exchange FM interaction is mediated via the Hund’s rule coupling between itinerant electrons and localized moments. A FM state occurs when the system has enough itinerant carriers^41^. In contrast, superexchange is an interaction which does not involve real carrier transfer. It mainly applies to the localized state^42^. In this scenario, both FM and AFM exchange can occur depending on orbital occupancy in accordance to Goodengough-Kanamori rule^42^,^43^. The valence of the Mn-ions in manganites is either +4 (Mn^4+^) or +3 (Mn^3+^). The large Hund’s coupling favors the population of the *t*_2g_ levels with three electrons, forming a spin 3/2 state, and the *e*_g_ level either contains one electron (Mn^3+^) or none (Mn^4+^). Double-exchange are mediated by the electrons at *e*_g_ orbital of Mn^3+^ ions, which is the only orbital contributing to the Fermi level. When hole doping is introduced, double-exchange induced ferromagnetism will be suppressed since the carrier density at the Fermi level decreases. With lost electron itinerancy, the superexchange interaction dominates, thus resulting in the change of magnetic ground state. Based on the Goodenough-Kanamori rule^42^,^43^, Mn^4+^-Mn^3+^ interactions are FM, while Mn^4+^-Mn^4+^ interactions are AFM. With the increase of the Mn^4+^/Mn^3+^ ratio, the ferromagnetism weakens progressively, from three dimensional FM to two dimensional FM (*i.e*. A-AFM), to one dimensional FM(C-AFM), and eventually to zero dimensional FM(G-AFM)). This explains the observation of the intermediate phases such as A-AFM, CE-AFM and C-AFM, which are characterized by FM interactions of one or two dimensions. Given the existence of competing FM and AFM interactions, it is not surprising to observe magnetic phase separation in some intermediate phases as indicated above. The above discussions also suggest that the FM metallic phase and the G-AFM insulating phase are far from each other in free energy.

Next let’s examine the ruthenates using the mechanism discussed above. First, the chemical valence of Ru ions is always 4+ in Ca_3_(Ru_1−*x*_Ti_*x*_)_2_O_7_ system. If only superexchange is taken into consideration, based on the Goodenough-Kanamori rule, the Ru^4+^-Ru^4+^ coupling should be AFM. One example is Ca_2_RuO_4_, which shows a G-AFM ground state^22^,^23^. The structure driven metal-to-insulator transition in this material provides an environment for the domination of superexchange interaction to take place. However, most of ruthenate compounds are itinerant. The superexchange interaction is submerged by the double-exchange interaction or other itinerant magnetisms. Ca_3_Ru_2_O_7_ also has a metal-to-insulator transition accompanied by a structural change as mentioned above^32^. Although the structure transition has a similar trend as that seen in Ca_2_RuO_4_, the space group does not change across the transition and the structural change is manifested only in the small changes of lattice parameters^32^. For this reason, below the MIT temperature (48 K), the Fermi surface is not fully gaped. ARPES measurements indeed proved the existence of small ungapped Fermi pockets^31^. This Fermi surface is about 2 orders of magnitude smaller than the Fermi surfaces seen in other metallic ruthenates such as Sr_2_RuO_4_ 44. Ca_3_Ru_2_O_7_ recovers and demonstrates metallic transport properties below ~30 K when the small Fermi pockets become coherent. Unlike manganites, Ca_3_Ru_2_O_7_ features comparable energy scales between the double-exchange FM and superexchange AFM interactions. The phase coherence of those small Fermi pockets is quite easy to be destroyed by Ti impurities, which are strong scattering centers^28^. Once those surviving itinerant electrons are localized, FM coupling strength would be reduced very quickly so that the checkboard super-exchange coupling becomes dominate, resulting in a G-AFM state. Our new phase diagram shown in Fig. 1a clearly demonstrates that the free energies of AFM-b and G-AFM states are close to each other, making the coexistence between AFM-b and G-AFM phases possible. What should be emphasized here is that the tuning parameter in our phase diagram is essentially the carrier itinerancy instead of band filling (holes doping) in manganites. The mechanism discussed here explains why the metallic AFM-b-to-insulating G-AFM transition can be triggered by a few percent Ti doping.

Besides the Ti-doping induced magnetic ground state transition from the AFM-b to the G-AFM phase in Ca_3_(Ru_1−*x*_Ti_*x*_)_2_O_7_, another noteworthy feature is the temperature driven magnetic phase transition, which happens within very narrow temperature ranges (below 20 K as shown in Fig. 1a). In Ca_3_Ru_2_O_7_, FM double exchange dominates in the whole temperature range below *T*_N_, resulting in AFM-a/AFM-b magnetic states. When Ti impurities are doped into Ru sites, it destroys the coherency of Fermi pockets as indicated above, resulting in charge carrier localization and double exchange FM interaction suppression. Such localization behavior enhances with temperature decreasing due to reduced thermal activation; this explains the evolution from the high-temperature AFM-a phase to the low-temperature G-AFM phase for 0.02 ≤ *x* < 0.05 (see Fig. 1a). The intermediate phase (IM) characterized by an incommensurate component between AFM-a and G-AFM^27^ is a consequence of the competition between the double-exchange FM and superexchange AFM interactions. It is interesting that the temperature ranges of AFM-a and IM phases shrink quickly with increasing Ti concentration and vanishes when *x* reaches 0.05. This further demonstrates that FM coupling strength, which depends on carrier itinerancy and plays an essential role in generating the high-temperature AFM-a phase, is extremely sensitive to Ti impurities. Given that chemical inhomogeneity is unavoidable for any doped systems, it is reasonable to expect some inhomogeneity in Ti distribution for the *x* = 0.02 −0.05 samples. Those local areas with richer Ti impurities favor the G-AFM ground state due to charge carrier localization caused by Ti impurities scattering, while for those local areas with less Ti impurities double exchange FM interaction can still survive, thus generating a AFM-b ground state. Therefore it is not surprising to observe the magnetic phase separation between G-AFM and AFM-b in the ground state for *x* = 0.02 −0.04.

In summary, we observed an evolution of magnetic phase separation near a magnetic phase boundary in the Ca_3_(Ru_1−*x*_Ti_*x*_)_2_O_7_ system. The G-AFM phase starts to appear for *x* = 0.02 as a minor phase and its volume fraction gradually increase with increasing Ti content. It becomes a dominant phase for *x* ≥ 0.03; eventually a pure G-AFM phase appears for *x* ≥ 0.05. These results, together with the observation of the evolution of AFM-a phase with temperature and Ti concentration, demonstrate that in Ca_3_Ru_2_O_7_, the double exchange FM interaction and AFM superexchange interaction between Ru ions have comparable energy scales. This makes the magnetic ground state of Ca_3_Ru_2_O_7_ extremely sensitive to impurity scattering such that a few percent Ti impurity doping can trigger a transition between two distinct magnetic ordered states, *i.e*. from AFM-b to G-AFM.

## Methods

Single crystals of Ca_3_(Ru_1−*x*_Ti_*x*_)_2_O_7_ used in this study were grown by floating zone technique. All samples used in our experiments were examined by X-ray diffraction (XRD) measurements and proven to be composed of pure bilayered phase. The successful doping of Ti into single crystals was confirmed by energy-dispersive x-ray spectroscopy (EDS). The real compositions are in general consistent with the nominal ones. XRD spectrum and EDS results are presented in supplementary Fig. S1 and Table S1 online. Magnetization measurements were performed with a superconducting quantum interference device (SQUID, Quantum Design) magnetometer. One important issue for magnetization measurements is twin domain, the presence of which would prevent the identification of spin-easy axis for an ordered magnetic state. To avoid this, samples for our magnetization measurements were carefully selected. The in-plane crystallographic directions were determined using Laue x-ray diffraction measurements. Every sample used the experiments was carefully examined by SQUID to ensure twin-domain free.

## Additional Information

**How to cite this article**: Peng, J. *et al*. Magnetic phase separation in double layer ruthenates Ca_3_(Ru_1−*x*_Ti*_x_*)_2_O_7_. *Sci. Rep*. **6**, 19462; doi: 10.1038/srep19462 (2016).

## Supplementary Material

Supplementary Information

## References

[b1] Bednorz J. G. K. A. M. possible high Tc superconductivity in the Ba-La-Cu-O system. Z. Phys. B: Condens. Matter 64, 189–193 (1986).

[b2] Anderson P. W. The Resonating Valence Bond State in La_2_CuO_4_ and Superconductivity. Science 235, 1196–1198 (1987). 17818979 10.1126/science.235.4793.1196

[b3] Ramirez A. P. Colossal magnetoresistance. J. Phys: Condens Mat 9, 8171 (1997).

[b4] Dagotto E., Hotta T. & Moreo A. Colossal magnetoresistant materials: The key role of phase separation. Phys. Rep. 344, 1–153 (2001).

[b5] Kusters R. M., Singleton J., Keen D. A., Mcgreevy R. & Hayes W. Magnetoresistance Measurements on the Magnetic Semiconductor Nd_0.5_Pb_0.5_MnO_3_. Physica. B 155, 362–365 (1989).

[b6] von Helmolt R., Wecker J., Holzapfel B., Schultz L. & Samwer K. Giant negative magnetoresistance in perovskitelike La_2/3_Ba_1/3_MnO_x_ ferromagnetic films. Phys. Rev. Lett. 71, 2331–2333 (1993). 10054646 10.1103/PhysRevLett.71.2331

[b7] Elbio D. Open questions in CMR manganites, relevance of clustered states and analogies with other compounds including the cuprates. New J. Phys. 7, 67 (2005).

[b8] Hein M. A. Progress, properties and prospects of passive high-temperature superconductive microwave devices in Europe. *Institute of Physics Conference Series*, *3rd European Conference on Applied Superconductivity (EUCAS 1997)*, Iop Publishing Ltd, **158,** 261–266, doi: 10.1088/0953-2048/10/12/001 (1997).

[b9] Withers R. S. & Ralston R. W. Superconductive Analog Signal-Processing Devices. Proc. Ieee. 77, 1247–1263 (1989).

[b10] Wolf S. A. . Spintronics: A Spin-Based Electronics Vision for the Future. Science 294, 1488–1495 (2001). 11711666 10.1126/science.1065389

[b11] Auciello O., Scott J. F. & Ramesh R. The physics of ferroelectric memories. Phys. Today 51, 22–27 (1998).

[b12] Scott J. F. Device Physics of Ferroelectric Memories. Ferroelectrics 183, 51–63 (1996).

[b13] Dagotto E. Complexity in Strongly Correlated Electronic Systems. Science 309, 257–262 (2005). 16002608 10.1126/science.1107559

[b14] Ishida K. . Spin-triplet superconductivity in Sr_2_RuO_4_ identified by 17O Knight shift. Nature 396, 658–660 (1998).

[b15] Mackenzie A. P. & Maeno Y. The superconductivity of Sr_2_RuO_4_ and the physics of spin-triplet pairing. Rev. Mod. Phys. 75, 657–712 (2003).

[b16] Nelson K. D., Mao Z. Q., Maeno Y. & Liu Y. Odd-Parity Superconductivity in Sr_2_RuO_4_. Science 306, 1151–1154 (2004). 15539595 10.1126/science.1103881

[b17] Grigera S. A. . Magnetic Field-Tuned Quantum Criticality in the Metallic Ruthenate Sr_3_Ru_2_O_7_. Science 294, 329–332 (2001). 11598292 10.1126/science.1063539

[b18] Perry R. S. . Metamagnetism and Critical Fluctuations in High Quality Single Crystals of the Bilayer Ruthenate Sr_3_Ru_2_O_7_. Phys. Rev. Lett. 86, 2661–2664 (2001). 11290005 10.1103/PhysRevLett.86.2661

[b19] Borzi R. A. . Formation of a Nematic Fluid at High Fields in Sr_3_Ru_2_O_7_. Science 315, 214–217 (2007). 17124288 10.1126/science.1134796

[b20] Ikeda S.-I., Maeno Y., Nakatsuji S., Kosaka M. & Uwatoko Y. Ground state in Sr_3_Ru_2_O_7_: Fermi liquid close to a ferromagnetic instability. Phys. Rev. B 62, R6089–R6092 (2000).

[b21] Klein L. . Anomalous Spin Scattering Effects in the Badly Metallic Itinerant Ferromagnet SrRuO_3_. Phys. Rev. Lett. 77, 2774–2777 (1996). 10062042 10.1103/PhysRevLett.77.2774

[b22] Nakatsuji S., Ikeda S.-I. & Maeno Y. Ca_2_RuO_4_: New Mott Insulators of Layered Ruthenate. J. Phys. Soc. Jpn. 66, 1868–1871 (1997).

[b23] Nakatsuji S. . Heavy-Mass Fermi Liquid near a Ferromagnetic Instability in Layered Ruthenates. Phys. Rev. Lett. 90, 137202 (2003). 12689321 10.1103/PhysRevLett.90.137202

[b24] Cao G., McCall S., Crow J. E. & Guertin R. P. Observation of a Metallic Antiferromagnetic Phase and Metal to Nonmetal Transition in Ca_3_Ru_2_O_7_. Phys. Rev. Lett. 78, 1751–1754 (1997).

[b25] Yoshida Y. . Quasi-two-dimensional metallic ground state of Ca_3_Ru_2_O_7_. Phys. Rev. B 69, 220411 (2004).

[b26] Cao G., McCall S., Shepard M., Crow J. E. & Guertin R. P. Thermal, magnetic, and transport properties of single-crystal Sr_1−x_Ca_x_RuO_3_ (0<~x<~1.0). Phys. Rev. B 56, 321–329 (1997).

[b27] Ke X. . Emergent electronic and magnetic state in Ca_3_Ru_2_O_7_ induced by Ti doping. Phys. Rev. B 84, 201102 (2011).

[b28] Peng J. . From quasi-two-dimensional metal with ferromagnetic bilayers to Mott insulator with G-type antiferromagnetic order in Ca_3_(Ru_1−x_Ti_x_)_2_O_7_. Phys. Rev. B 87, 085125 (2013).

[b29] Lee J. S. . Pseudogap Dependence of the Optical Conductivity Spectra of Ca_3_Ru_2_O_7_: A Possible Contribution of the Orbital Flip Excitation. Phys. Rev. Lett. 98, 097403 (2007). 17359196 10.1103/PhysRevLett.98.097403

[b30] Liu H. L., Yoon S., Cooper S. L., Cao G. & Crow J. E. Raman-scattering study of the charge and spin dynamics of the layered ruthenium oxide Ca_3_Ru_2_O_7_. Phys. Rev. B 60, R6980–R6983 (1999).

[b31] Baumberger F. . Nested Fermi Surface and Electronic Instability in Ca_3_Ru_2_O_7_. Phys. Rev. Lett. 96, 107601 (2006). 16605788 10.1103/PhysRevLett.96.107601

[b32] Yoshida Y. . Crystal and magnetic structure of Ca_3_Ru_2_O_7_. Phys. Rev. B 72, 054412 (2005).

[b33] Bao W., Mao Z. Q., Qu Z. & Lynn J. W. Spin Valve Effect and Magnetoresistivity in Single Crystalline Ca_3_Ru_2_O_7_. Phys. Rev. Lett. 100, 247203 (2008). 18643623 10.1103/PhysRevLett.100.247203

[b34] Mitchell J. F. . Spin, Charge, and Lattice States in Layered Magnetoresistive Oxides. J. Phys. Chem. B 105, 10731–10745 (2001).

[b35] Kajimoto R. . Hole-concentration-induced transformation of the magnetic and orbital structures in Nd_1−*x*_Sr_*x*_MnO_3_. Phys. Rev. B 60, 9506–9517 (1999).

[b36] Papavassiliou G. . ^55^Mn NMR Investigation of Electronic Phase Separation in La_1−*x*_Ca_*x*_MnO_3_ for 0.2 ≤ *x *≤ 0.5. Phys. Rev. Lett. 84, 761–764 (2000). 11017366 10.1103/PhysRevLett.84.761

[b37] Papavassiliou G. . Polarons and phase separation in lanthanum-based manganese perovskites: A ^139^La and ^55^Mn NMR study. Phys. Rev. B 59, 6390–6394 (1999).

[b38] Qu Z. . Unusual heavy-mass nearly ferromagnetic state with a surprisingly large Wilson ratio in the double layered ruthenates (Sr_1−x_Ca_x_)_3_Ru_2_O_7_. Phys. Rev. B 78, 180407 (2008).

[b39] Lin X. N., Zhou Z. X., Durairaj V., Schlottmann P. & Cao G. Colossal Magnetoresistance by Avoiding a Ferromagnetic State in the Mott System Ca_3_Ru_2_O_7_. Phys. Rev. Lett. 95, 017203 (2005). 16090650 10.1103/PhysRevLett.95.017203

[b40] Peng J. . Interplay between the lattice and spin degrees of freedom in (Sr_1−x_Ca_x_)_3_Ru_2_O_7_. Phys. Rev. B 82, 024417 (2010).

[b41] Zener C. Interaction between the *d*-Shells in the Transition Metals. II. Ferromagnetic Compounds of Manganese with Perovskite Structure. Phys. Rev. 82, 403–405 (1951).

[b42] Goodenough J. B. Theory of the Role of Covalence in the Perovskite-Type Manganites [La, *M*(II)]MnO_3_. Phys. Rev. 100, 564–573 (1955).

[b43] Kanamori J. Superexchange interaction and symmetry properties of electron orbitals. J. Phys. Chem. Solids 10, 87–98 (1959).

[b44] Puchkov A. V., Shen Z. X., Kimura T. & Tokura Y. ARPES results on Sr_2_RuO_4_: Fermi surface revisited. Phys. Rev. B 58, R13322–R13325 (1998).

