# Supplementary material for: Magnetic phase separation in double layer ruthenates Ca3(Ru1−xTix)2O7
Source: Sci Rep. 2016 Jan 14;6:19462. doi: 10.1038/srep19462 (PMC4725874; doi:10.1038/srep19462)
Supplement: Supplementary Information [file srep19462-s1.pdf]

Supplementary Information for

Magnetic phase separation in double layer ruthenates $Ca_3(Ru_{1-x}Ti_x)_2O_7$

Jin Peng, [1, 2] J. Y. Liu, [2] J. Hu, [2] Z. Q. Mao, [2*] F. M. Zhang[1] and X. S. Wu [1*]

[1] *Collaborative Innovation Center of Advanced Microstructures, Lab of Solid State Microstructures, School of Physics, Nanjing University, Nanjing 210093, P. R. China*

[2] *Department of Physics and Engineering Physics, Tulane University, New Orleans, Louisiana 70118, USA*

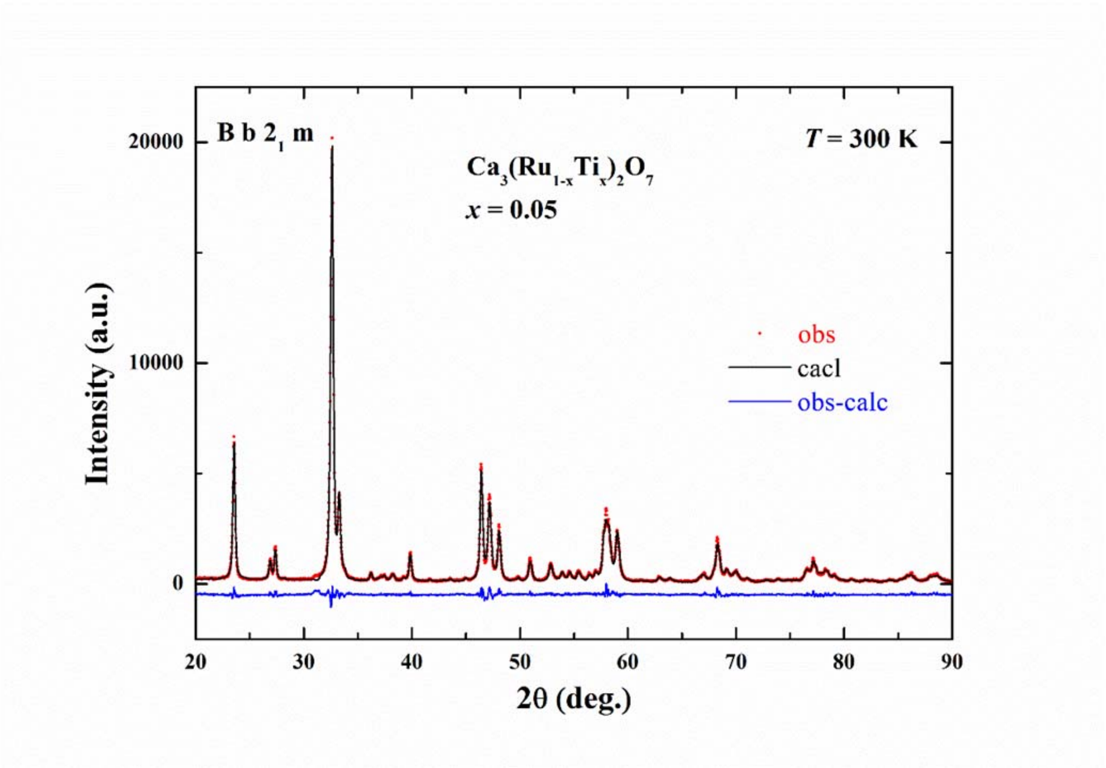

SI-Fig. S1: X-ray diffraction pattern of 5% Ti doped $Ca_3Ru_2O_7$ together with corresponding calculated profile using the *Bb*2$_1$*m* space group. XRD pattern proved our samples to be composed of pure bilayered phase with the same space group symmetry as pristine compound $Ca_3Ru_2O_7$.

Sample / Composition	$x = 0.02$	$x = 0.03$	$x = 0.04$	$x = 0.05$
EDS	$0.0163 \leq x \leq 0.0210$	$0.0276 \leq x \leq 0.0334$	$0.0378 \leq x \leq 0.0430$	$0.0459 \leq x \leq 0.0519$

SI-Table. S1: Energy Dispersive Spectrometer (EDS) results of $Ca_3(Ru_{1-x}Ti_x)_2O_7$. The successful doping of Ti into single crystals can be confirmed by EDS spectroscopy.
